# Inhibition of PCSK9 does not improve lipopolysaccharide-induced mortality in mice[S]

**DOI:** 10.1194/jlr.M076844

**Published:** 2017-06-09

**Authors:** Jean-Mathieu Berger, Angel Loza Valdes, Jesper Gromada, Norma Anderson, Jay D. Horton

**Affiliations:** Departments of Internal Medicine and Molecular Genetics* University of Texas Southwestern Medical Center, Dallas, TX; Center for Human Nutrition,§ University of Texas Southwestern Medical Center, Dallas, TX; Regeneron Pharmaceuticals, Inc.,† Tarrytown, NY

**Keywords:** lipopolysaccharide, endotoxemia, cholesterol, LDL, PCSK9

## Abstract

Proprotein convertase subtilisin/kexin type 9 (PCSK9) is a secreted protein that targets LDL receptors (LDLRs) for degradation in liver. Blocking the interaction of PCSK9 with the LDLR potently reduces plasma LDL cholesterol levels and cardiovascular events. Recently, it has been suggested that inhibition of PCSK9 might also improve outcomes in mice and humans with sepsis, possibly by increasing LDLR-mediated clearance of endotoxins. Sepsis is a complication of a severe microbial infection that has shared pathways with lipid metabolism. Here, we tested whether anti-PCSK9 antibodies prevent death from lipopolysaccharide (LPS)-induced endotoxemia. Mice were administered PCSK9 antibodies prior to, or shortly after, injecting LPS. In both scenarios, the administration of PCSK9 antibodies did not alter endotoxemia-induced mortality. Afterward, we determined whether the complete absence of PCSK9 improved endotoxemia-induced mortality in mice with the germ-line deletion of *Pcsk9*. Similarly, PCSK9 knockout mice were not protected from LPS-induced death. To determine whether low LDLR expression increased LPS-induced mortality, *Ldlr^−/−^* mice and PCSK9 transgenic mice were studied after injection of LPS. Endotoxemia-induced mortality was not altered in either mouse model. In a human cohort, we observed no correlation between plasma inflammation markers with total cholesterol levels, LDL cholesterol, and PCSK9. Combined, our data demonstrate that PCSK9 inhibition provides no protection from LPS-induced mortality in mice.

Proprotein convertase subtilisin/kexin type 9 (PCSK9) is a circulating protein secreted primarily from the liver that binds and degrades LDL receptors (LDLRs) mainly in the liver (1, 2). Gain-of-function mutations in *PCSK9* are associated with hypercholesterolemia and increased cardiovascular disease (3). Conversely, loss-of-function (LOF) mutations in *PCSK9* are associated with low plasma LDL cholesterol levels and reduced cardiovascular events (4). Human monoclonal antibodies targeting circulating PCSK9 have been developed that block its interaction with the LDLR and reduce plasma LDL cholesterol levels. Several phase II and phase III clinical trials in hypercholesterolemic individuals administered anti-PCSK9 antibodies as monotherapy, or in addition to statins, reduced both plasma LDL cholesterol levels by 45%–60% (5–9) and cardiovascular events (10, 11).

Although PCSK9 clearly is an important regulator of LDL cholesterol (12), little is known about whether PCSK9 may have other detrimental or beneficial actions. Recently, studies have suggested that PCSK9 potentiates sepsis-induced mortality (13–16). Sepsis is a systemic infection that results in generalized inflammation that can lead to organ failure and death. Currently, there are no specific treatments for sepsis other than antibiotics (17). Sepsis alters cholesterol metabolism by reducing reverse cholesterol transport (18), a vital pathway by which lipopolysaccharides (LPSs) are cleared and detoxified from the body (19, 20). LPS is a component of gram-negative bacterial cell walls and binds to serum proteins, including LDL and HDL (21). Thus, in response to inflammation, LPS is taken up by the liver and excreted from the body via bile (22). Previously, Feingold et al. (16) administered LPS to mice and reported an ∼60% decrease in hepatic LDLR protein. The increase in LDLRs was attributed to the increase in PCSK9 expression that was also observed following LPS injection. The induction of PCSK9 mRNA expression was found at very low levels of LPS and increased in a concentration-dependent manner (16).

Inasmuch as LPS can be cleared via the LDLR, it is possible that alterations in LDLR expression could influence the clinical consequences of sepsis. Statins increase expression levels of LDLRs, and beyond their cholesterol lowering effect, statins may have pleiotropic properties including anti-inflammation, immunomodulation, and improved endothelial function with reduced apoptosis. Statins reduce the production of proinflammatory cytokines known to be detrimental in the development and progression of sepsis. However, whether individuals with severe infections on statins have better outcomes is still under debate. Since the first prospective observational population-based study was published in 2004 (23), numerous additional observational studies have reported an association between statins and a reduced risk of infectious outcomes such as pneumonia, sepsis, and infection-related mortality (24–27). In contrast, in randomized placebo-controlled trials of statins in critically ill patients with severe sepsis, statins failed to provide a survival benefit (28–31). Several meta-analyses of randomized clinical trials showed no effect of statins on the reduced risk of infection-related death; therefore, the likelihood of a causal effect as reported in observational studies is unlikely (32–34).

PCSK9 degrades LDLRs; thus it is also possible that PCSK9 could alter sepsis outcomes through its potent regulation of the LDLR or through other as yet undiscovered mechanisms. Recent studies in a polymicrobial sepsis mouse model using cecal ligation and puncture (CLP) demonstrated that repeated injections of an antibody against PCSK9 in addition to antibiotics were able to decrease the inflammatory response and increase survival (13). In humans, plasma PCSK9 concentrations increased in a group of septic patients, which correlated with sepsis-induced cardiovascular or respiratory failure (14).

Here, we determined whether alirocumab, an anti-PCSK9 antibody, improved survival in mice that were administered LPS. The administration of alirocumab either before or after LPS injection did not alter LPS-induced mortality. Similarly, *Pcsk9^−/−^* mice were not protected from LPS-induced death. Last, in a human cohort we found no correlation of plasma inflammation markers with total cholesterol, LDL cholesterol, or PCSK9 concentrations.

## MATERIALS AND METHODS

### Materials

Alirocumab, an anti-PCSK9 antibody directed against human PCSK9 (35), and REGN1932, a control antibody, were provided by Regeneron (NY). Additional anti-PCSK9 antibodies were purchased from BPS Bioscience (CA) and control antibodies from R&D Systems (MN). LPS (from smooth type *Escherichia coli* strain K58 and *Pseudomonas aeruginosa*, purified by phenol extraction) was obtained from Sigma (MO).

### Animals

C57Bl/6J wild-type and *Ldlr^−/−^* mice were purchased from Jackson Laboratories (ME). *Pcsk9^+/−^* mice were used to produce *Pcsk9^+/+^* and *Pcsk9^−/−^* mice (36). Transgenic mice expressing human PCSK9 in the liver (Tg-hPCSK9) on a *Pcsk9^−/−^* background were generated, as has been described (1). Human cholesteryl ester transfer protein (CETP) Tg-ApoB100 mice were described previously (37). Mice were housed under standard conditions with free access to food and water under a 12-h light/12-h dark cycle in a temperature-controlled environment. Mice were fed a standard rodent chow diet (2018 chow diet; Harlan). All experiments were conducted using 10- to 11-week-old male mice. The institutional animal care and use committee of the University of Texas Southwestern Medical Center approved all experimental procedures (APN 2015-100863).

### Plasma analytes

Total plasma cholesterol concentrations were determined using an enzymatic kit from Thermo Fisher Scientific (MA). Plasma cytokines concentrations [interleukin-6 (IL-6), IL-10, macrophage inflammatory protein 2 (MIP-2), and TNFα] were measured with Multiplex Assays using Luminex technology from MilliporeSigma (Darmstadt, Germany).

### LPS-induced septic death survival studies

LPS (1, 5, 7.5, 10, 15, or 20 mg/kg) was injected intraperitoneally to induce a systemic inflammatory response. When testing the effect of an anti-PCSK9 antibody, LPS was injected prior to or after the antibody administration, as is described in the figure legends. Mice were monitored every hour for the first 12 h and then every 2 h for the next 72 h, and time of death was recorded. In all studies, blood was drawn 48 h before any intervention to determine basal plasma metabolite concentrations.

### Human studies

Human plasma samples were obtained from the Dallas BioBank collection (38). Patients in that cohort are well characterized and have been screened for known *PCSK9* mutations. Briefly, total cholesterol was measured with an enzymatic assay, and LDL cholesterol concentrations were estimated using the Friedewald equation (38). PCSK9 concentrations in the plasma were measured with an ELISA, as has been described previously (39). Plasma cytokines levels (IL-6, IL-8, IL-10, growth-regulated oncogene α, and TNFα) were determined by Multiplex Assays using Luminex technology from MilliporeSigma (Darmstadt, Germany).

### Statistics

All results are reported as means ± SEM. Statistical significance was analyzed using a nonparametric Mann-Whitney test or a log-rank Mantel-Cox test. The values of *P* < 0.05 were considered as significant.

## RESULTS

### Alirocumab fails to protect mice from LPS-induced death

We first established the minimal lethal dose of LPS by performing a dose-response experiment in wild-type C57Bl/6J mice (supplemental Fig. S1). In the same range of doses that has been previously described (19, 40), we found that 20 mg/kg was the lowest dose of LPS that induced the highest mortality rate in the shortest period of time. We also established that the dose of 10 mg/kg of alirocumab maximally reduced plasma cholesterol levels in mice (data not shown).

**Fig. 1.**
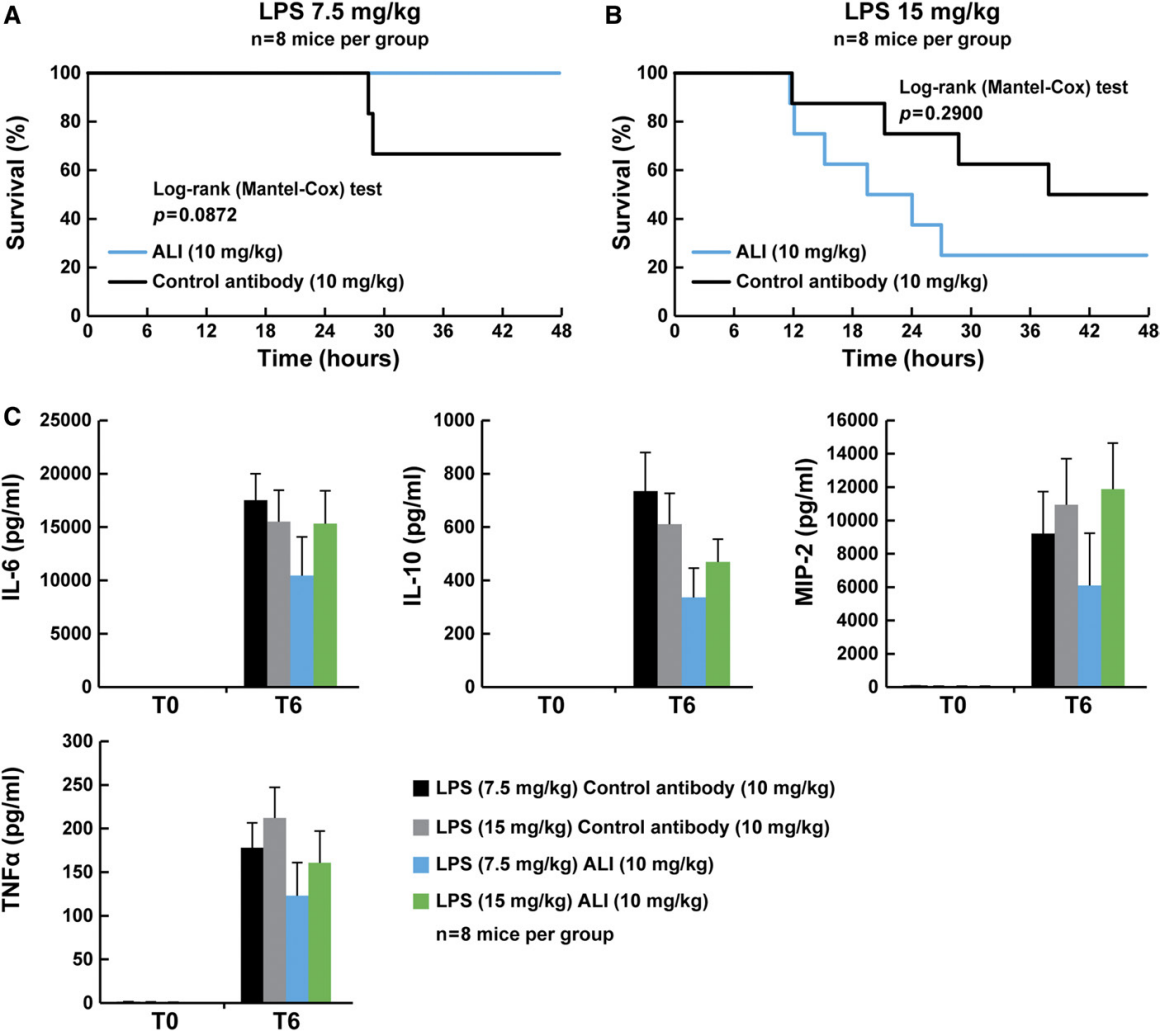
Administration of an anti-PCSK9 antibody does not reduce LPS-induced mortality. Survival curves of C57Bl/6J wild-type mice injected with control antibody (REGN1932, 10 mg/kg, sc) or alirocumab (10 mg/kg, sc) after LPS administration (n = 8 per group). Antibodies are injected 2 h after LPS inoculation: 7.5 mg/kg, ip (A), and 15 mg/kg, ip (B) at time 0. Mice were monitored hourly for 72 h and time of death recorded. C: Plasma inflammation markers in C57Bl/6J wild-type mice (n = 8 per group) treated with control antibody (REGN1932, 10 mg/kg, sc) or alirocumab (10 mg/kg, sc) before and 6 h after LPS (7.5 or 15 mg/kg, ip). All values represent means ± SEM. ALI, alirocumab; NS, no significant difference.

To determine whether inhibition of PCSK9 could be used therapeutically to improve outcomes of endotoxemia, C57Bl/6J male mice were injected with 7.5 or 15 mg/kg of LPS, and 2 h later alirocumab (10 mg/kg, ip) was administered. As is shown in **Fig. 1A, B**, there was no reduction in mortality in mice that received alirocumab. Similar results were obtained in two independent experiments (data not shown). We also examined inflammatory markers before and 6 h after LPS injections and found no significant changes in IL-6, IL-10, MIP-2, or TNFα in mice treated with the anti-PCSK9 antibody (Fig. 1C).

**Fig. 2.**
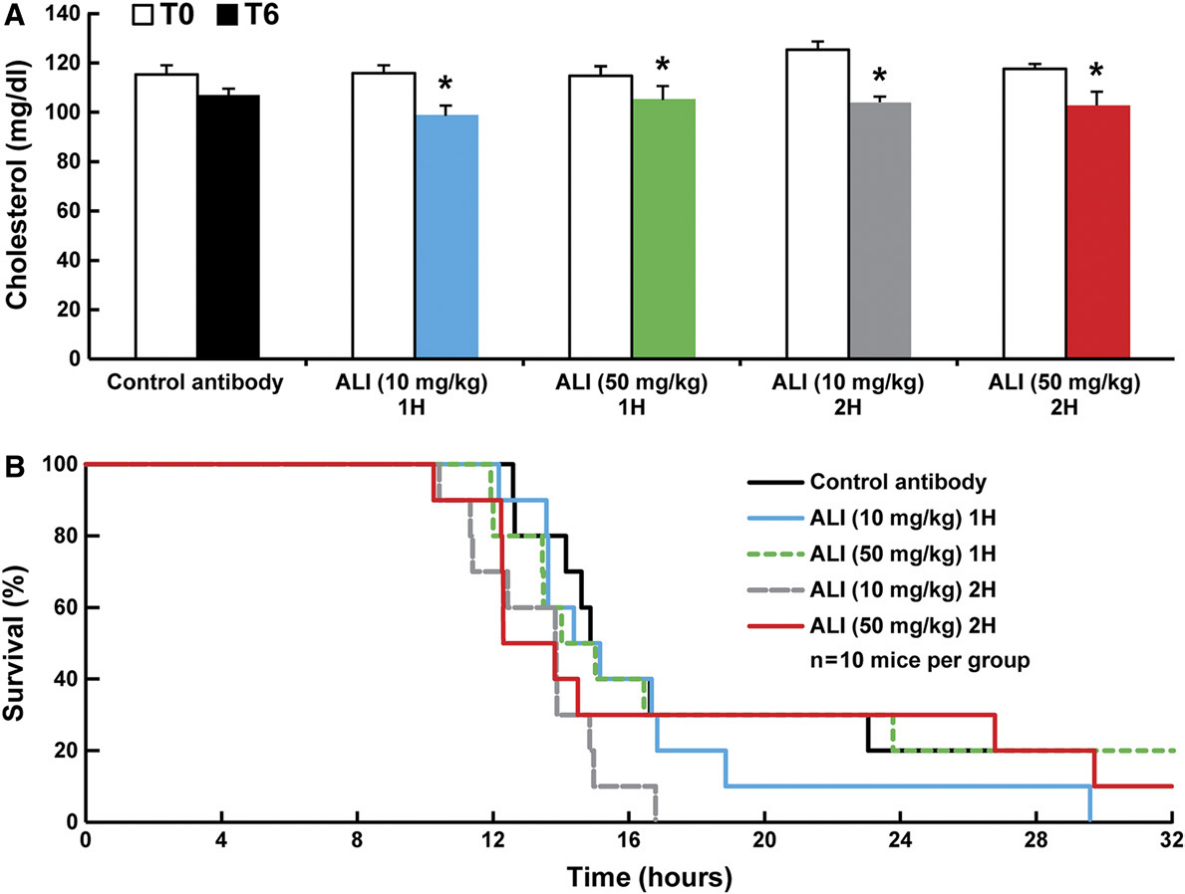
Alirocumab administered at various times does not change LPS-induced mortality. A: Plasma cholesterol levels of C57Bl/6J wild-type mice (n = 10 per group) treated with control antibody (REGN1932, 50 mg/kg, iv) or alirocumab (10 or 50 mg/kg, iv) before and 6 h after LPS (20 mg/kg, ip). Both doses of alirocumab were injected 1 or 2 h after LPS inoculation. All values represent means ± SEM. *P < 0.05. B: Survival curves of C57Bl/6J wild-type mice (n = 10 per group) injected with control antibody (REGN1932, 50 mg/kg, iv) or alirocumab (10 or 50 mg/kg, iv) 1 or 2 h after LPS (20 mg/kg, ip, time 0). Mice were monitored hourly for 72 h, and time of death was recorded.

Next, we determined whether the mode of alirocumab antibody administration could improve the response to endotoxemia. For these experiments, alirocumab or control REGN1932 (10 or 50 mg/kg) was injected intravenously 1 or 2 h after LPS inoculation of C57Bl/6J male mice. Plasma cholesterol levels were similarly reduced with both doses of alirocumab (**Fig. 2A**). With either dose of alirocumab, LPS-induced mortality was not altered (Fig. 2B).

As is discussed above, LPS can bind LDL and can be cleared by the LDLR. Unlike humans, mice normally have very low LDL cholesterol concentrations. Therefore, we determined whether blocking PCSK9 action could alter LPS-induced mortality in a mouse model with high levels of LDL cholesterol and normal functioning LDLRs. For this purpose, we used transgenic mice that express CETP and ApoB100 (TgCETP;ApoB100), which have a plasma lipoprotein profile closer to that found in humans (37). TgCETP;ApoB100 mice were injected with control antibody (REGN1932, 10 mg/kg, sc) or alirocumab, 10 mg/kg, sc) 2 h after LPS inoculation. The administration of alirocumab did not improve survival following the injection of 7.5 or 15 mg/kg of LPS (supplemental Fig. S2A, B).

**Fig. 3.**
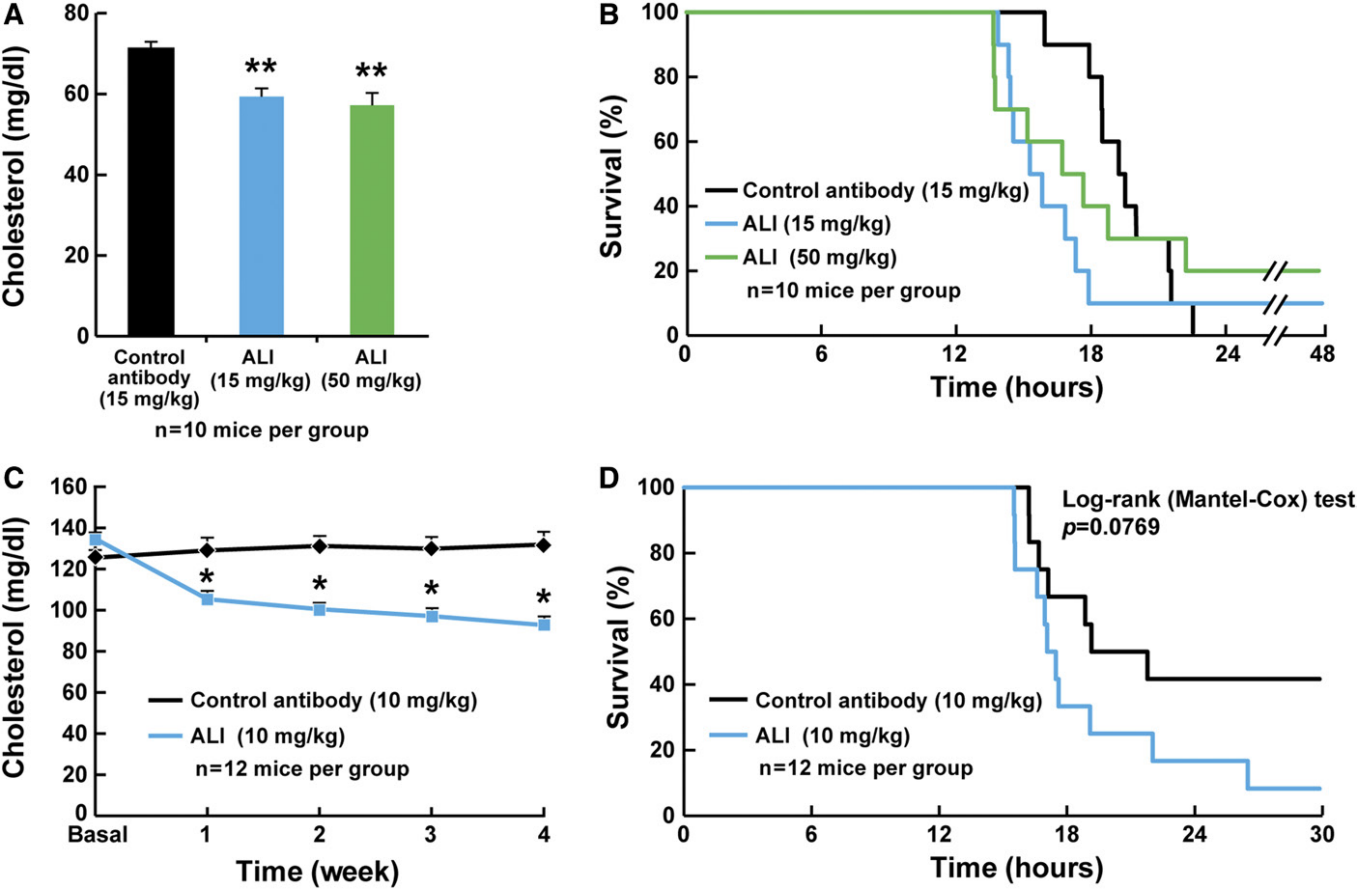
Pretreatment with an anti-PCSK9 antibody does not protect mice from LPS-induced death. A: Plasma cholesterol levels 48 h after administration of a control antibody (REGN1932, 15 mg/kg, sc) or alirocumab (15 or 50 mg/kg, sc) injected into C57Bl/6J mice (n = 10 per group). Values represent means ± SEM. **P < 0.01. B: Survival curves of C57Bl/6J mice injected with LPS (20 mg/kg, ip) 48 h after the administration of the control antibody (REGN1932, 15 mg/kg, sc) or alirocumab (15 or 50 mg/kg, sc) (n = 10 per group). Mice are checked hourly for 72 h and time of death recorded. C: Plasma cholesterol levels of C57Bl/6J mice injected with control antibody (REGN1932, 10 mg/kg, sc) or alirocumab (10 mg/kg, sc) every week for 4 weeks (n = 12 per group). All values represent means ± SEM. *P < 0.05. D: Survival curves of C57Bl/6J mice injected with control antibody (REGN1932, 10 mg/kg, sc) or alirocumab (10 mg/kg, sc). LPS (20 mg/kg, ip) was injected (time 0) 48 h after the last antibody injection. Mice were monitored hourly for 72 h, and time of death was recorded.

We next determined whether blocking PCSK9 with alirocumab antibody could prevent LPS-induced mortality. C57Bl/6J male mice were injected with 15 or 50 mg/kg of alirocumab or control REGN1932 48 h before LPS administration. Plasma cholesterol levels were reduced to the same extent (∼20%) with 15 or 50 mg/kg of alirocumab 48 h after administration (**Fig. 3A**). As is shown in Fig. 3B, pretreatment with alirocumab did not reduce LPS-induced mortality. To exclude the possibility that blocking PCSK9 action could be effective at lower LPS loads, mice were injected with alirocumab (10 mg/kg), followed by LPS at concentrations of 7.5 and 15 mg/kg (supplemental Fig. S3). Plasma cholesterol was again reduced by 20% 48 h after treatment with alirocumab (supplemental Fig. S3A). However, alirocumab again failed to provide protection from LPS-induced death when compared with the control antibody (supplemental Fig. S3B, C).

Finally, we tested whether long-term PCSK9 inhibition could protect mice from LPS-induced death. C57Bl/6J mice were injected weekly for a month with the control antibody or alirocumab (10 mg/kg). Plasma cholesterol was reduced by 20% with alirocumab, and this reduction remained constant over the course of the 4 weeks of treatment (Fig. 3C). As is shown in Fig. 3D, survival after administration of LPS in mice chronically treated with alirocumab was not significantly different from those that were administered the control antibody. Similar results were obtained in two independent experiments.

### Use of a mouse anti-PCSK9 antibody fails to improve survival after LPS-induced mortality

**Fig. 4.**
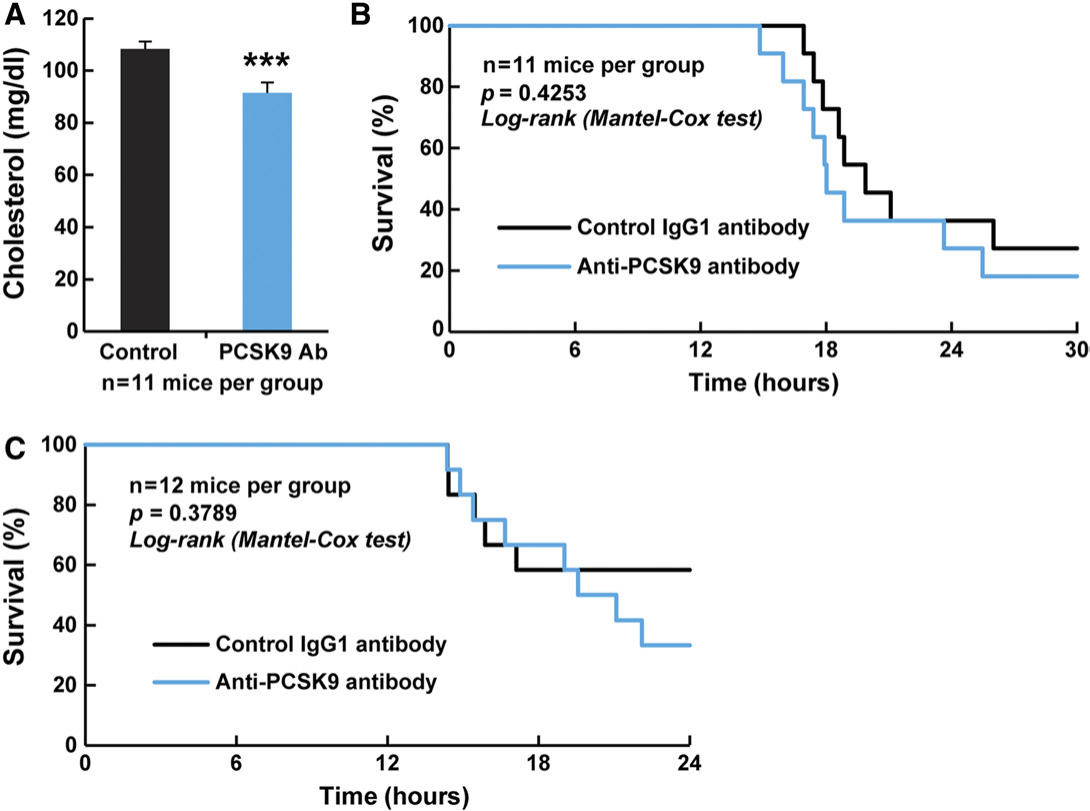
PCSK9 antibody directed against mouse PCSK9 fails to improve LPS-induced mortality. A: Plasma cholesterol levels in C57Bl/6J mice (n = 11 per group) 48 h after control antibody (IgG1 MAB002, R&D Systems; 100 µg/mouse, sc) or anti-PCSK9 antibody (71207, BPS Bioscience; 100 µg/mouse, sc) injection. All values represent means ± SEM. ***P < 0.001. B: Survival curves in C57Bl/6J mice (n = 11 per group) injected with control antibody (IgG1 MAB002, R&D Systems; 100 µg/mouse, sc) or anti-PCSK9 antibody (71207, BPS Bioscience, 100 µg/mouse, sc). Antibodies were injected 48 and 1 h prior to LPS (20 mg/kg, ip, time 0). Mice were checked hourly for 72 h, and time of death was recorded. C: Survival curves of C57Bl/6J mice (n = 12 per group) injected with control antibody (IgG1 MAB002, R&D Systems) or anti-PCSK9 antibody (71207, BPS Bioscience; 100 µg/d/mouse, sc, 6 h after LPS (20 mg/kg, ip, time 0). Mice were monitored hourly for 72 h, and time of death was recorded.

To confirm that the negative results obtained above were not due to the use of alirocumab, which is directed against human PCSK9, we repeated the studies using the commercially available anti-PCSK9 antibody previously used for PCSK9 inhibition in mice (13). Mice were first injected with the anti-PCSK9 antibody (71207, BPS Bioscience; 100 µg/mouse, sc) 48 or 1 h before the injection of LPS (20 mg/kg). Similar to the results obtained by the administration of alirocumab, plasma cholesterol concentrations were reduced by ∼20% in mice given the 71,207 antibody (**Fig. 4A**). As is shown in Fig. 4B, pretreatment with the anti-PCSK9 antibody again did not protect mice from LPS-induced septic death. Similarly, administration of the anti-PCSK9 antibody 6 and 24 h after LPS administration did not reduce mortality (Fig. 4C).

### *Pcsk9^−/−^* mice are not protected from LPS-induced mortality

**Fig. 5.**
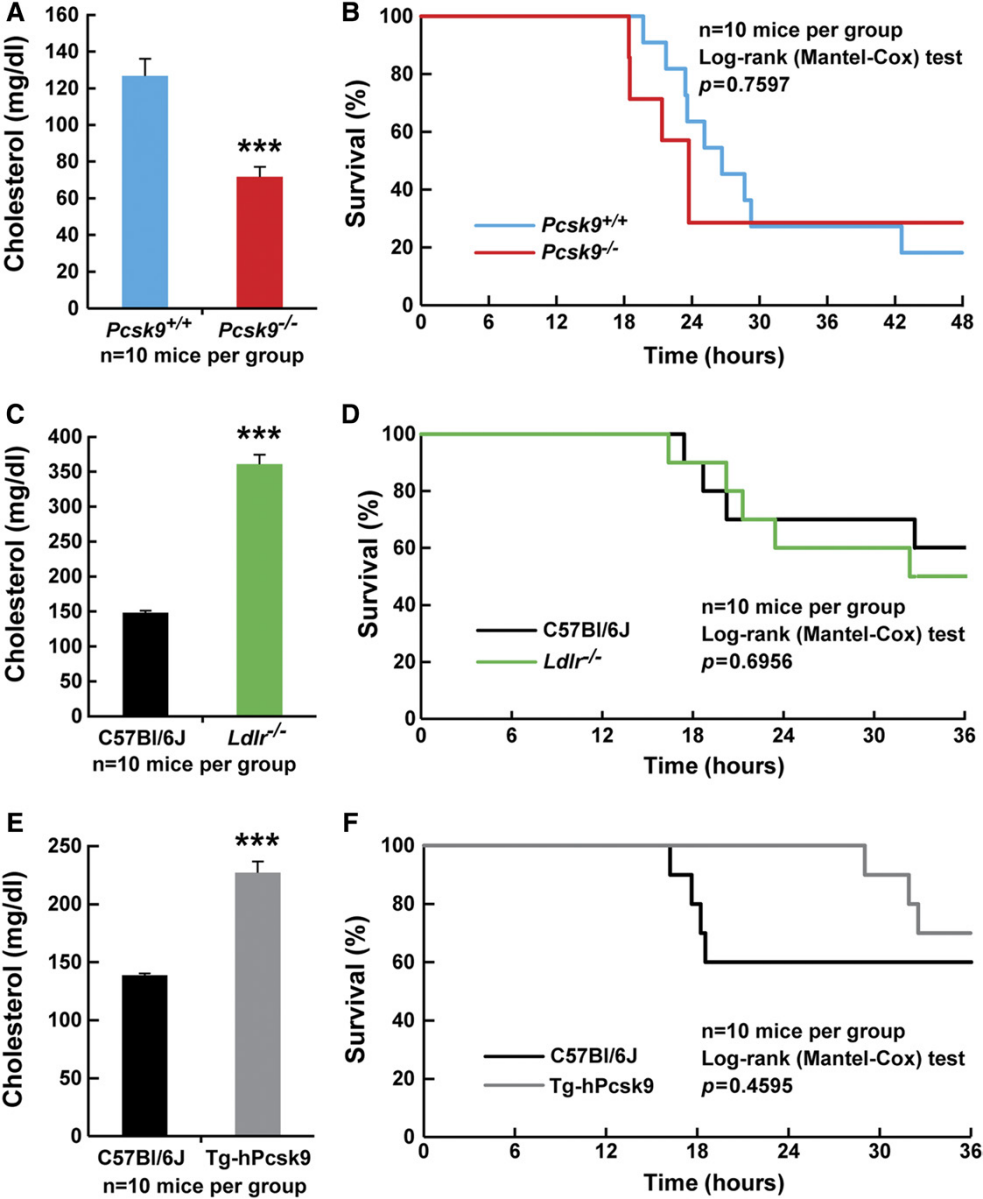
Altering LDLR levels does not change LPS-induced mortality. A, C, and E: Plasma cholesterol levels of C57Bl/6J, *Pcsk9^+/+^*,* Pcsk9^−/−^*,* Ldlr^−/−^*, and Tg-hPCSK9 mice before LPS injection (n = 10 per group). All values represent means ± SEM. ***P < 0.001. B, D, and F: Survival curves of C57Bl/6J, *Pcsk9^+/+^*,* Pcsk9^−/−^*,* Ldlr^−/−^*, and Tg-hPCSK9 mice (n = 10 per group) after LPS injection (20 mg/kg, ip, time 0). Mice were monitored hourly for 72 h, and time of death was recorded.

Finally, we repeated studies in *Pcsk9^−/−^* mice to confirm that PCSK9 does not play a direct role in protecting mice from LPS-induced death (36). *Pcsk9^−/−^* mice have significantly lower plasma cholesterol concentrations compared with their *Pcsk9^+/+^* control counterparts (**Fig. 5A**). Mice were injected with LPS from *E. coli* (20 mg/kg, ip), and time of death was recorded for the following 72 h. As is shown in Fig. 5B, LPS-injected *Pcsk9^−/−^* mice had similar survival profiles compared with the LPS-injected *Pcsk9^+/+^* control mice. We validated this result with a second source of LPS from *P. aeruginosa* and also found that *Pcsk9^−/−^* mice were not protected from LPS-induced mortality (supplemental Fig. S4A, B).

### Lack of LDLRs does not increase LPS-induced mortality

The above studies do not directly eliminate a possible role of the LDLR in LPS-induced mortality. To test the hypothesis that LDLRs may alter LPS-induced death through its ability to participate in LPS clearance, we used the *Ldlr^−/−^* and Tg-hPCSK9 mice, which have very low levels of LDLR expression due to high levels of PCSK9 (Fig. 5C, E). No change of the survival rates was observed in either *Ldlr^−/−^* and Tg-hPCSK9 mice compared with wild-type C57Bl/6J mice when challenged with LPS (Fig. 5D, F).

### Inflammation markers in a human cohort do not correlate with plasma total cholesterol levels, LDL cholesterol, and PCSK9 concentrations

**Fig. 6.**
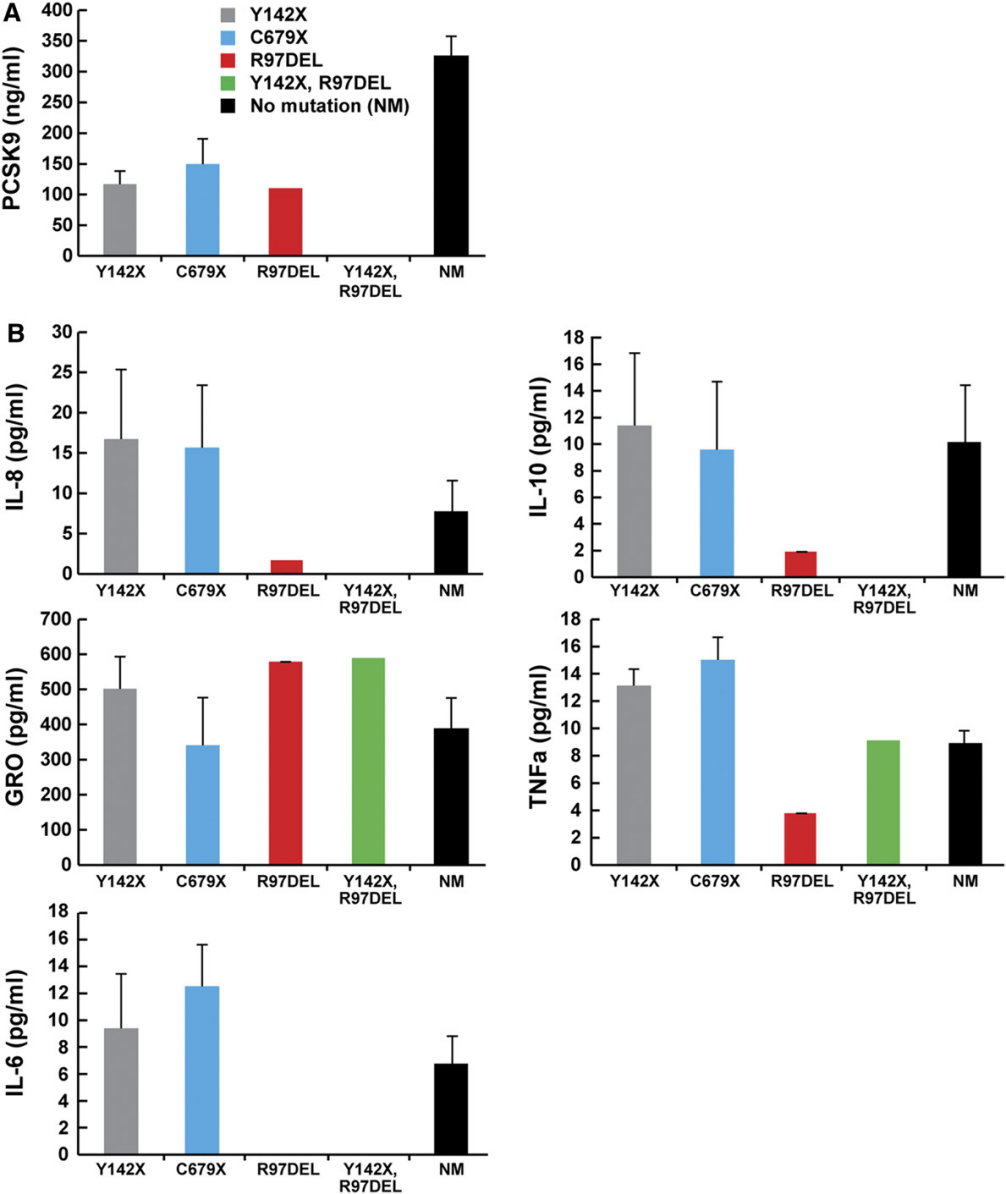
No correlation between plasma inflammation markers and PCSK9 LOF mutations in humans. Concentrations of plasma PCSK9 (A) and cytokine levels (B) in subjects with *PCSK9* LOF mutations (n = 28; six Y142X, four C679X, one R97DEL, one Y142X/R97DEL, and 16 with no *PCSK9* mutation). All values represent means ± SEM. NM, no mutation.

To determine whether we could establish a correlation between inflammation markers and PCSK9 in plasma, we measured the concentrations of selected pro- and anti-inflammatory cytokines, PCSK9, total cholesterol, and LDL cholesterol in 28 plasma samples from the Dallas BioBank collection (38). Sixteen of these samples were obtained from patients with no known mutations in *PCSK9*, and 12 were from patients with known LOF mutations in *PCSK9*. In this set of samples, we validated the positive correlation between plasma PCSK9 and both LDL cholesterol and total cholesterol (supplemental Fig. S5A, B). No correlation was found with five different markers of inflammation and total cholesterol (supplemental Fig. S5C), LDL cholesterol (supplemental Fig. S5D), or PCSK9 (supplemental Fig. S5E). PCSK9 LOF patients had lower plasma PCSK9 concentrations (**Fig. 6A**), but no significant difference in concentration of inflammatory markers compared with samples from control patients (Fig. 6B).

## DISCUSSION

Clearance of LPS requires binding to transfer proteins and transport through lipoproteins, including LDL and HDL. Thus, increasing the flux through these pathways could be beneficial in individuals with endotoxemia. Inhibition of PCSK9 increases the expression of LDLRs in liver, which leads to enhanced LDL cholesterol clearance in mice (36). Similarly, studies in humans administered anti-PCSK9 antibodies have a reduction in plasma LDL cholesterol by up to 70%, again most likely through increased LDL cholesterol clearance from the blood (35). Therefore, we tested the hypothesis that increasing clearance of LDL using an anti-PCSK9 antibody could protect mice from LPS-induced death. Here we show that in a mouse model of endotoxemia induced by LPS administration, preventive or curative therapeutic approaches using a monoclonal antibody directed against PCSK9 at doses more than 10× higher than those used in humans did not reduce LPS-induced death (Figs. 1, 3, 5).

A previous study by Walley et al. (13) found that *Pcsk9^−/−^* mice had lower cytokine levels compared with wild-type mice following a challenge with LPS. They also reported that *Pcsk9^−/−^* mice had reduced systemic and cardiovascular responses to LPS treatment. Dwivedi et al. (15) found the same reduced inflammation and increased protection against CLP-induced sepsis in *Pcsk9^−/−^* mice. Unfortunately, our studies in *Pcsk9^−/−^* mice and in wild-type mice treated with an anti-PCSK9 antibody could not replicate these positive results in the LPS-induced septic death model.

Walley et al. (13) used an antibiotic, imipenem, injected subcutaneously twice daily in addition to an anti-PCSK9 antibody treatment to mimic clinical conditions. To test whether anti-PCSK9 antibodies as a monotherapy have a direct impact on endotoxemia and to rule out any potential synergic effect of treatment combinations, we did not use antibiotics. We also used, in all our experiments, an intraperitoneal injection of LPS from gram-negative bacteria to induced endotoxemia. In comparison with the CLP technique, LPS injection is less invasive and induces less interindividual variability. This different approach may explain why our results do not show any improvement of survival when PCSK9 is inhibited in mice administered LPS.

One hypothesis as to why inhibition of PCSK9 might protect mice from LPS-induced death is through the reduced degradation of the LDLR and thus enhancing LPS clearance. Our results do not support this hypothesis inasmuch as *Pcsk9^−/−^* mice, which have an ∼2.8-fold increase in LDLR protein expression in liver and enhanced LDL-cholesterol clearance (36), were not protected from LPS-induced death. Conversely, mice with the genetic absence of LDLRs or very low levels of LDLR expression as a result of high PCSK9 expression were not more susceptible to LPS-induced death. Thus, it does not appear that the LDLR plays a discernable role in this mouse model of endotoxemia.

In a retrospective study in humans, SNPs in *PCSK9* correlated with survival after septic shock (13). Here, we show in healthy subjects with or without PCSK9 LOF mutations, plasma inflammation markers do not correlate with total cholesterol, LDL cholesterol, or plasma PCSK9 levels.

In summary, we provide evidence that anti-PCSK9 antibodies alone do not protect mice from LPS-induced death. Similarly, we found no direct role for the LDLR in LPS-induced mortality. Whether PCSK9, the LDLR, or both may be important in the response to polymicrobial sepsis was not directly tested in these studies and will require further investigation.

## Supplementary Material

Supplemental Data
