# Supplementary material for: Inhibition of PCSK9 does not improve lipopolysaccharide-induced mortality in mice
Source: J Lipid Res. 2017 Jun 9;58(8):1661–9. doi: 10.1194/jlr.M076844 (PMC5538287; doi:10.1194/jlr.M076844)
Supplement: Supplemental Data [file supp_58_8_1661__index.html]

Inhibition of PCSK9 does not improve lipopolysaccharide-induced mortality in mice — Inhibition of PCSK9 does not improve lipopolysaccharide-induced mortality in mice — Supplemental Data 

# Inhibition of PCSK9 does not improve lipopolysaccharide-induced mortality in mice

## Supplemental Data

- Supplemental Figures (.pdf, 947 KB) - Supplemental information
