# Supplementary material for: Inhibition of PCSK9 does not improve lipopolysaccharide-induced mortality in mice
Source: J Lipid Res. 2017 Jun 9;58(8):1661–9. doi: 10.1194/jlr.M076844 (PMC5538287; doi:10.1194/jlr.M076844)
Supplement: Supplemental Data [file 10.1194_M076844_jlr.M076844-1.pdf]

SUPPLEMENTAL INFORMATION:

Inhibition of PCSK9 does not improve lipopolysaccharide-induced mortality in mice

Jean-Mathieu Berger[1], Angel Loza Valdes[1], Jesper Gromada[3], Norma Anderson[1] and Jay D. Horton[1,2]

[1] Departments of Internal Medicine and Molecular Genetics, University of Texas Southwestern Medical Center, Dallas, TX, USA

[2] Center for Human Nutrition, University of Texas Southwestern Medical Center, Dallas, TX, USA

[3] Regeneron Pharmaceuticals, Inc., Tarrytown, NY 10591, USA

Fig. S1

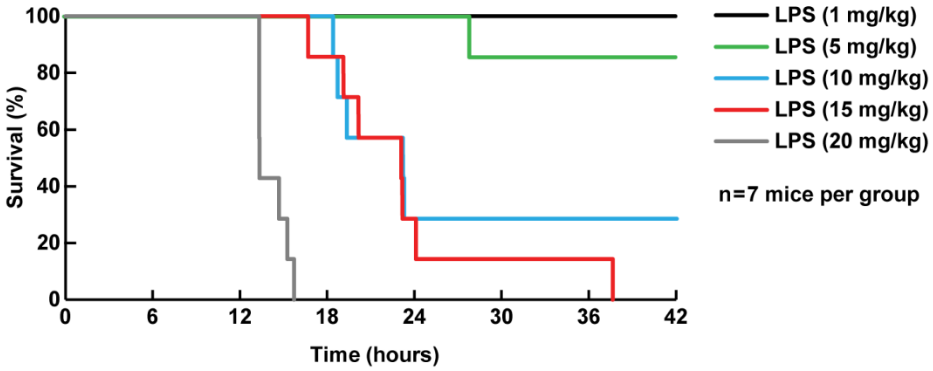

Supplemental Fig. S1. LPS dose-response in C57Bl/6J mice. Survival curves in C57Bl/6J mice (n=7 per group) injected with 1, 5, 10, 15, or 20 mg/kg LPS (I.P., time 0). Mice were monitored hourly for 72 h and time of death recorded.

Fig. S2

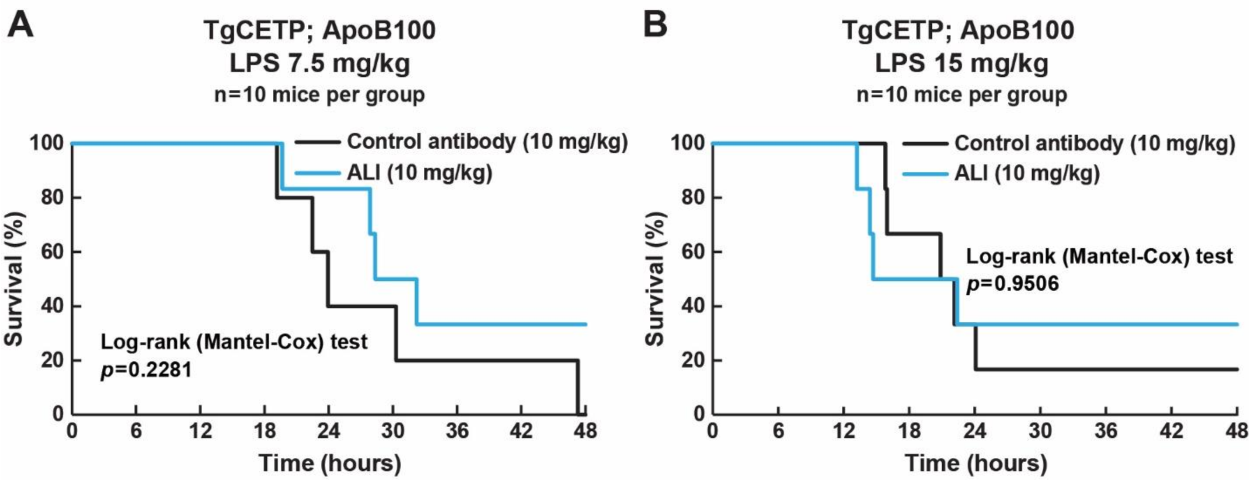

Fig. S2. Anti-PCSK9 antibody administration following various doses of LPS in TgCETP;ApoB100 mice. Survival curves of TgCETP;ApoB100 mice (n=10 per group) injected with control antibody (REGN1932, 10 mg/kg S.C.) or alirocumab (ALI) (10 mg/kg S.C.) 2 h after LPS administration (7.5 mg/kg (A) and 15 mg/kg (B) I.P., time 0). Mice were monitored hourly for 72 h and time of death recorded.

Fig. S3

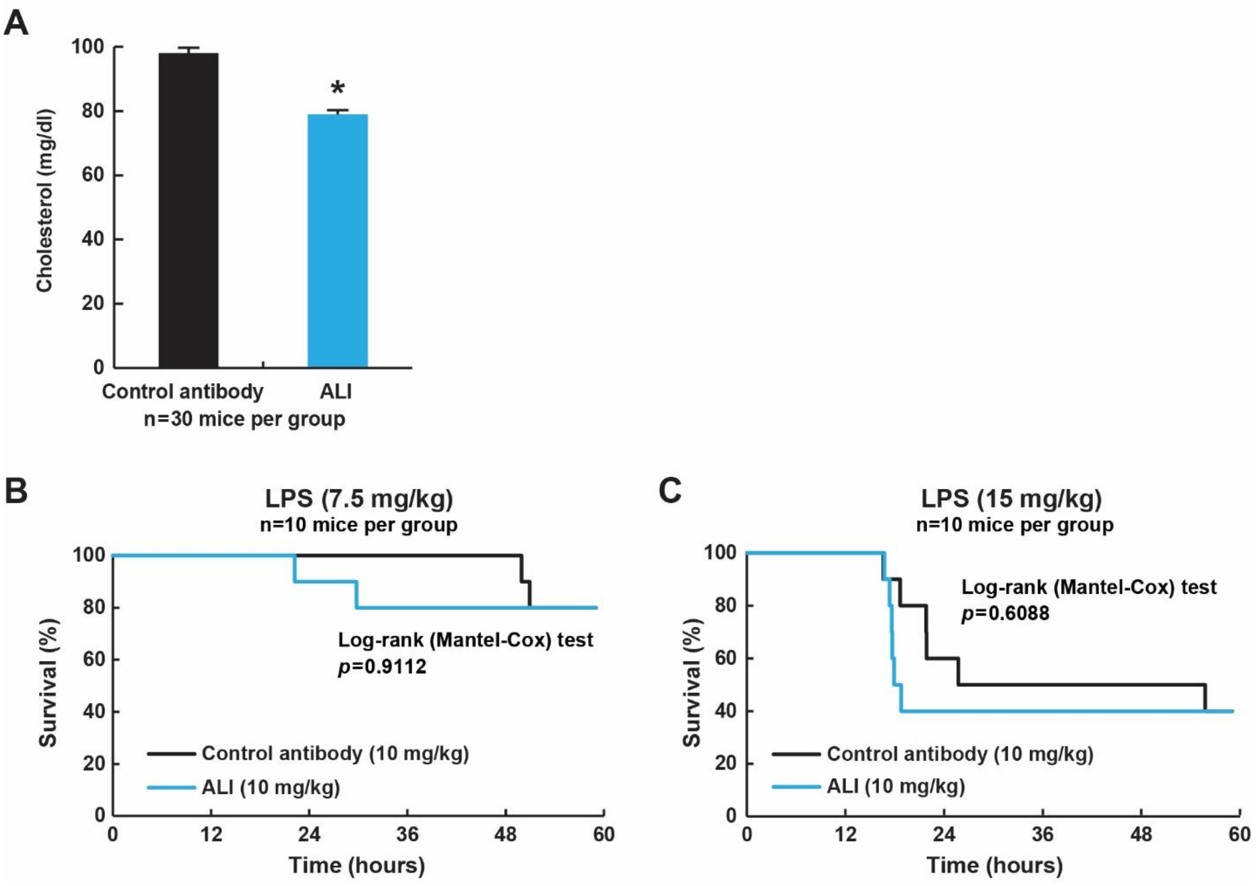

Fig. S3. Pre-treatment with an anti-PCSK9 antibody does not alter LPS-induced mortality. (A) Plasma cholesterol levels in C57Bl/6J wild-type mice (n=30 per group) 48 h after control antibody (REGN1932, 10 mg/kg S.C.) or alirocumab (ALI) (10 mg/kg S.C.) injection. All values represent means ± SEM. * $p<0.05$. Survival curves of C57Bl/6J mice (n=10 per group) injected with LPS (7.5 mg/kg (B) and 15 mg/kg (C) I.P.) 48 h after antibody administration. Mice were monitored hourly for 72 h and time of death recorded.

Fig. S4

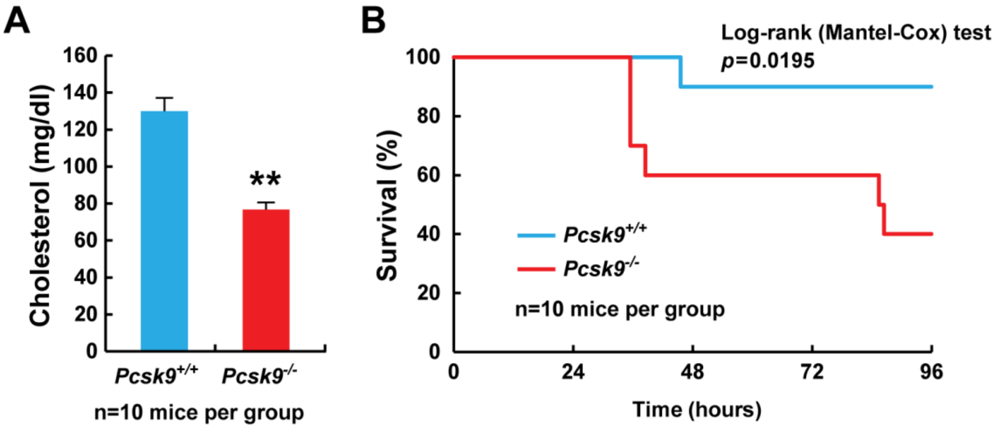

Fig. S4. *Pcsk9*$^{-/-}$ mice are not protected from *P. aeruginosa* LPS-induced death. (A) Plasma cholesterol levels of *Pcsk9*$^{+/+}$ and *Pcsk9*$^{-/-}$ mice before LPS administration (n=10 per group). All values represent means ± SEM. ** p<0.01. (B) Survival curves of *Pcsk9*$^{+/+}$ and *Pcsk9*$^{-/-}$ mice (n=10 per group) following LPS (20 mg/kg I.P.) administration. Mice were monitored hourly for 72 h and time of death recorded.

Fig. S5

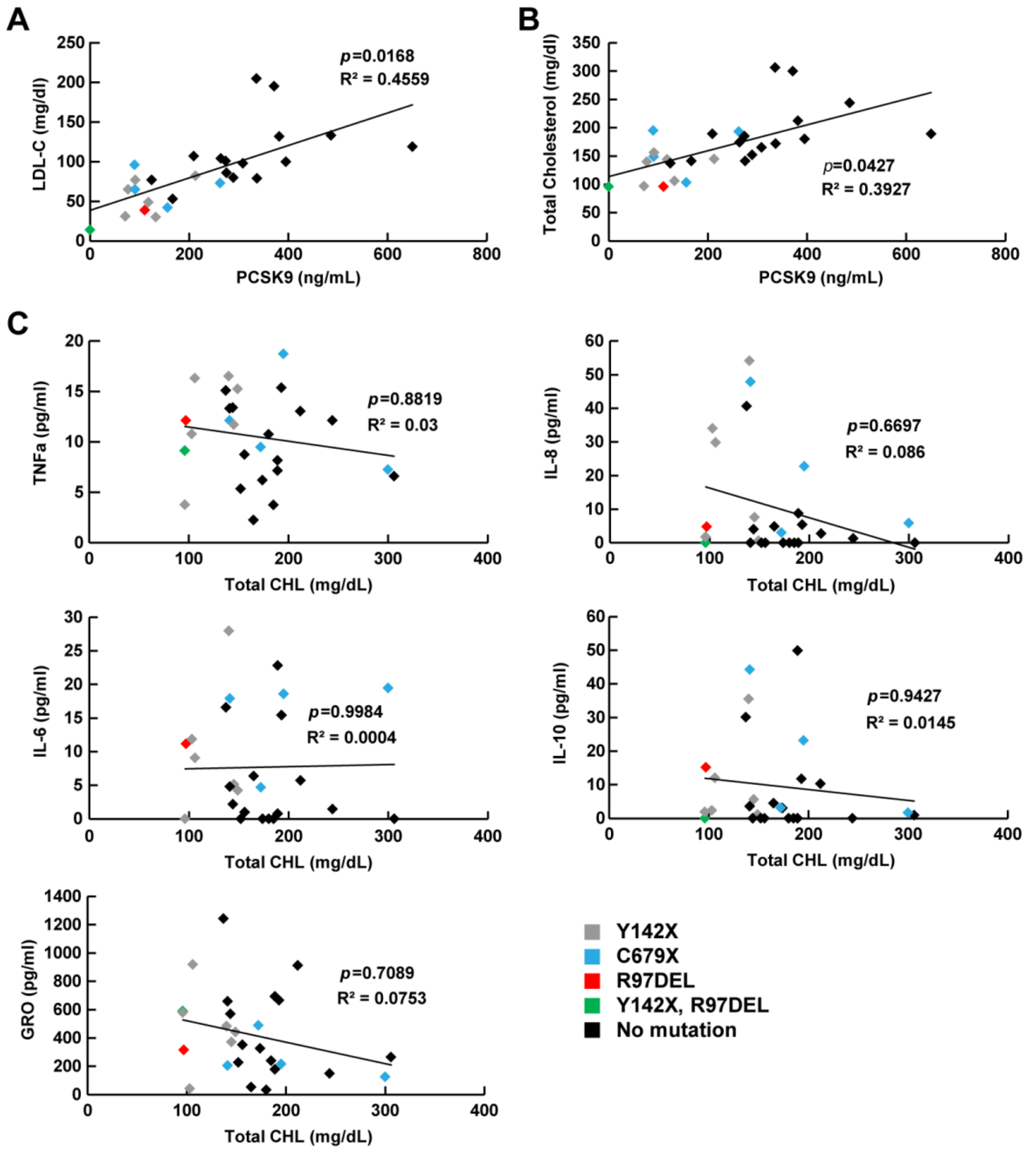

A
LDL-C (mg/dl)
PCSK9 (ng/mL)
p=0.0168
R² = 0.4559
B
Total Cholesterol (mg/dl)
PCSK9 (ng/mL)
p=0.0427
R² = 0.3927
C
TNFa (pg/ml)
Total CHL (mg/dL)
p=0.8819
R² = 0.03
IL-8 (pg/ml)
p=0.6697
R² = 0.086
IL-6 (pg/ml)
p=0.9984
R² = 0.0004
IL-10 (pg/ml)
p=0.9427
R² = 0.0145
GRO (pg/ml)
p=0.7089
R² = 0.0753
Y142X
C679X
R97DEL
Y142X, R97DEL
No mutation

Fig. S5

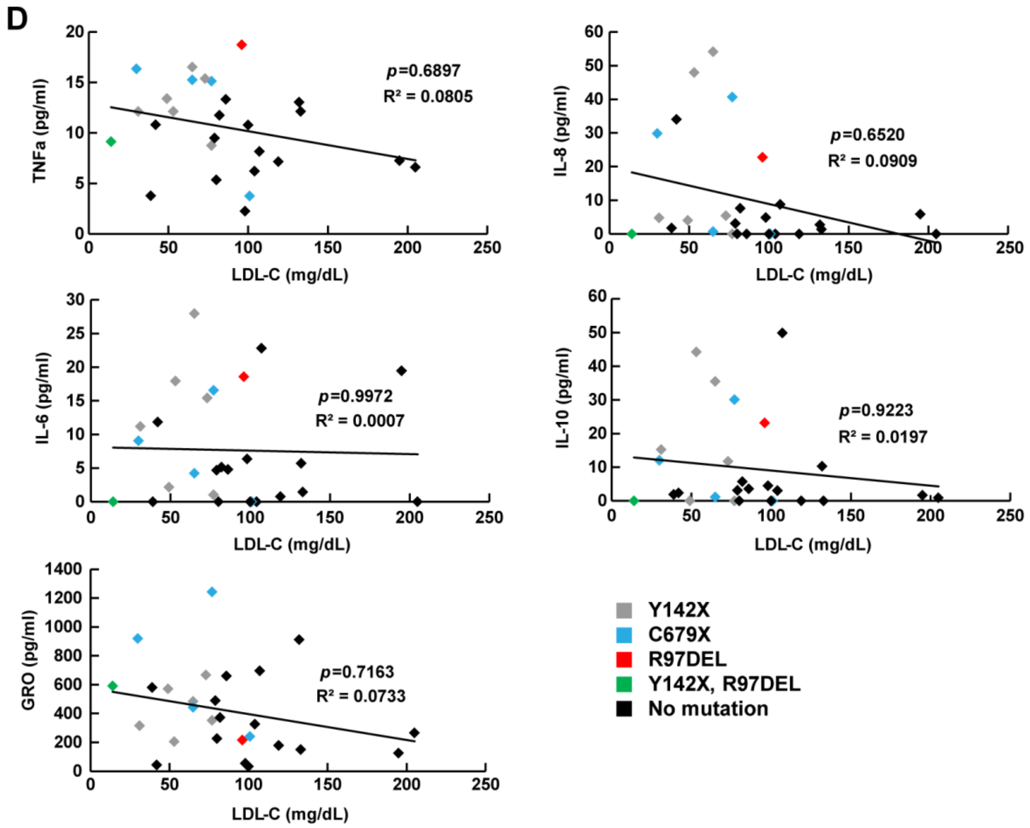
D
TNFa (pg/ml)
p=0.6897
R² = 0.0805
LDL-C (mg/dL)
IL-8 (pg/ml)
p=0.6520
R² = 0.0909
LDL-C (mg/dL)
IL-6 (pg/ml)
p=0.9972
R² = 0.0007
LDL-C (mg/dL)
IL-10 (pg/ml)
p=0.9223
R² = 0.0197
LDL-C (mg/dL)
GRO (pg/ml)
p=0.7163
R² = 0.0733
LDL-C (mg/dL)
Y142X
C679X
R97DEL
Y142X, R97DEL
No mutation

Fig. S5

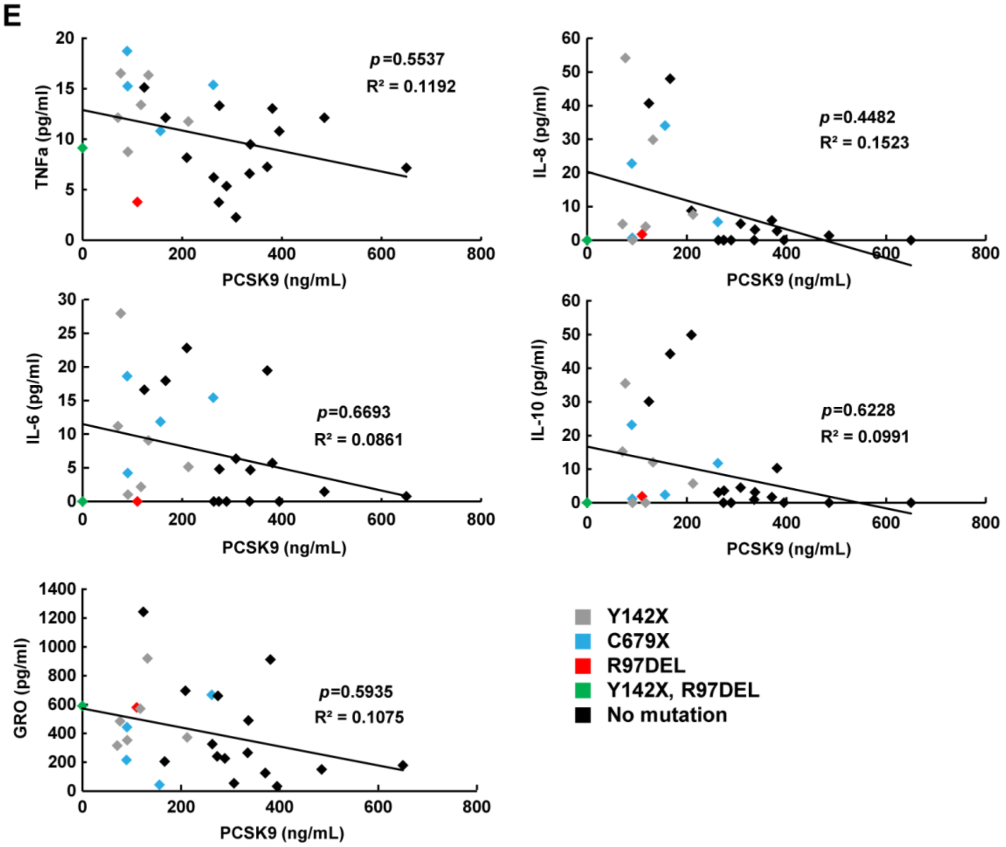

Fig. S5. No correlation between plasma inflammation markers and total cholesterol, LDL-cholesterol (LDL-C) and PCSK9 concentrations in human. Correlation of human PCSK9 plasma levels with LDL-C (A) and total cholesterol (B). Correlation of total plasma cholesterol (C), LDL-C (D) and PCSK9 (E) with inflammation markers in human (n=28 patients; 6 Y142X, 4 C679X, 1 R97DEL, 1 Y142X/R97DEL, 16 with no *PCSK9* mutation). All values represent means ± SEM.
